# Occupational burnout among doctors at Mankweng and Pietersburg hospitals, Limpopo province

**DOI:** 10.4102/safp.v65i1.5745

**Published:** 2023-09-28

**Authors:** Hlayisani V. Mamorobela, Gert J.O. Marincowitz, Clara Marincowitz

**Affiliations:** 1 Department of Family Medicine, Faculty of Health Sciences, University of Limpopo, Mankweng, South Africa; 2 Limpopo Health, Mankweng Hospital, Mankweng, South Africa; 3 Department of Family Medicine, Limpopo Department of Health, Mankweng Hospital, Mankweng, South Africa; 4 Department of Psychiatry, Faculty of Health Sciences, University of Stellenbosch, Tygerberg, South Africa; 5 SA Medical Research Council, Cape Town, South Africa

**Keywords:** prevalence, burnout, occupational burnout, healthcare worker, healthcare professional, doctor, hospital, Limpopo

## Abstract

**Background:**

The purpose of this study was to assess the presence of occupational burnout among full-time employed doctors of all ranks at the Mankweng and Pietersburg tertiary academic hospitals in South Africa’s Limpopo province.

**Methods:**

A quantitative, observational study was conducted firstly to determine whether burnout was present among medical doctors at these institutions and, secondly, to quantify the amount of burnout in those affected. Data collection was done using structured questionnaires. All ranks of medical doctors from various departments participated in the study, resulting in a total sample size of 150.

**Results:**

The study revealed that occupational burnout was present at these institutions, with an overall prevalence of 36%. When compared to other studies conducted at public sector hospitals in South Africa, this figure appears to fall within the middle range. However, different studies have used different criteria to measure burnout.

**Conclusion:**

Currently, there is too much variation in the criteria of burnout among different studies, making comparisons difficult. More studies are needed to standardise the measurement of burnout.

**Contribution:**

The main contribution of the research is to understand the extent of burnout at the tertiary hospital in Limpopo province.

## Introduction

Occupational burnout among medical doctors is a major concern globally.^1^ Burnout can be described as a job-related stress syndrome caused by chronic exposure to work stress.^1^ Work environments with excessive work schedules and high demands, plus the need to prove that one’s value, leave employees feeling emotionally drained, cynical about their work, and with a low sense of personal accomplishment (PA).^1^ Physical depletion, feelings of helplessness, negative self-concept and negative attitudes towards work, life and others follow.^2^ Additionally, new technologies such as mobile devices can exacerbate burnout by preventing disconnection and the necessary recovery from work.^3^

The concept of burnout was first described in 1981^2^ and has since been refined to include three major components, namely emotional exhaustion (EE), depersonalisation (DP) and reduced PA.^2^ Emotional exhaustion is defined as a state of emotional and sometimes physical depletion. Work overload and personal conflicts in interpersonal relationships were shown to be some of the causes of EE^4^. Depersonalisation refers to negative and cynical attitudes towards one’s clients or patients, or towards work in general.^5^ The third and final aspect of burnout, reduced PA, refers to the tendency to doubt the meaning and quality of one’s work.^6^

There have been a few studies conducted on burnout in the South African public medical context. Among rural hospital doctors in the Western Cape, 81% of participants had high EE or DP scores.^7^ Similarly, in the Cape Metropole, 76% of public sector doctors also experienced burnout.^8^ In Bloemfontein, however, overall prevalence was much lower, with 26.3% of public sector doctors experiencing burnout.^9^ It is not clear why such a great variation exists between these two settings; one possible explanation is that it could be because of discrepancies in the criteria used to define and measure burnout in different studies.^10^

Despite some research being available, the understanding of burnout, especially among doctors working in the public sector in Limpopo province, is still limited. The lack of standardisation makes it difficult to form an accurate picture of burnout in the South African public medical context.^3^ To understand this problem better among full-time doctors at the two tertiary institutions in the Limpopo province, the prevalence and some associated factors were investigated.

## Methodology

A quantitative observational cross-sectional study was conducted in 2017 among all medical doctors working at Mankweng Hospital (32 km to the east of Polokwane, the capital of Limpopo province) and Pietersburg Hospital (situated in Polokwane). Both hospitals employed 382 doctors of all ranks, including consultants (specialists), registrars, medical officers and medical interns. No community service doctors were included in the study because at that stage Limpopo province did not place community service doctors at the tertiary hospitals. All doctors working at these institutions, except part-time employed doctors, doctors doing locums, maxillo-facial surgeons and forensic pathologists, qualified for inclusion.

### Data collection

The Maslach Burnout Inventory (MBI) is the most widely used and validated tool for measuring burnout.^3^ The MBI measures all three burnout dimensions (EE, DP and PA) using 7-point Likert scales indicating the frequency of characteristic symptoms. Final scores are then classified either as low range, moderate or high range (see Table 1).^11^ For the purposes of this research, burnout was regarded as a high-range score in the EE and/or DP dimensions (see Table 1). Personal accomplishment is greatly dependent on resources.^11^ Because resources are frequently a problem in public sector, it was excluded in this study to assess burnout.

**TABLE 1:** Maslach burnout inventory classification of burnout.

Category of burnout	Low range	Moderate range	High range
Emotional exhaustion (EE)	0–16	17–26	≥ 27
Depersonalisation (DP)	0–6	7–12	≥ 13
Personal accomplishment (PA)^†^	≥ 39	32–38	0–31

PA, personal accomplishment.

† The value of PA is inversely related to burnout. Thus, a lack of PA is a part of the burnout syndrome.

In questionnaire-based researches, bias may be introduced by the questionnaire itself. For example, when it comes to research topics that contain buzzwords such as ‘burnout’, participants could gravitate towards answers that they feel will satisfy the researcher, if the hypothesis is made known to them.^12^ To avoid this, our questionnaire was labelled as a ‘job satisfaction survey’, as suggested by Maslach et al.^11^

The questionnaire was piloted at the Mankweng Hospital, Department of Family Medicine and 26 doctors completed the questionnaire. Subsequently, clarifications were added to improve the user-friendliness of the questionnaire. The responses obtained from piloting were also included in the final study dataset because the required number of participants could not be reached. After this, voluntary participation in the final survey occurred as the researcher had time to go to the meetings over a 10-month period from August 2018 to May 2019. The researcher attended the departmental meetings of the other departments in the hospitals and requested doctors to participate. Participants were provided with adequate instructions prior to filling in their questionnaires manually. The sample size was calculated as 194 with the Yamane formula.^13^ A hundred and fifty participants were recruited to complete the questionnaire, 77.7% of the required number.

### Data analysis

The data were analysed using statistical software Statistical Package for Social Sciences (SPSS) 25.0. The demographic data were described and summarised. The prevalence of burnout was correlated with socio-demographic variables. A Chi-square test was used to determine whether a significant relationship exists between burnout and the participants’ demographics. Statistical significance was reported on a 95% confidence interval. The significance level (*p* < 0.05) was used as a guideline to determine significant relationships.

## Results

Questionnaires were completed by 150 doctors of all ranks from both hospitals. Of these participants, 95 (63.0%) were working in Mankweng Hospital during the time of data collection and 55 (37.0%) were working in Pietersburg Hospital. Of the total, 69 (46.0%) were men and 81 (54.0%) were women. The breakdown by rank was as follows: intern doctors and medical doctors constituted 37% (56) each, followed by registrars (18 [12.0%]) and specialists (20 [13.4%]).

### Respondents’ level of burnout

**FIGURE 1:**
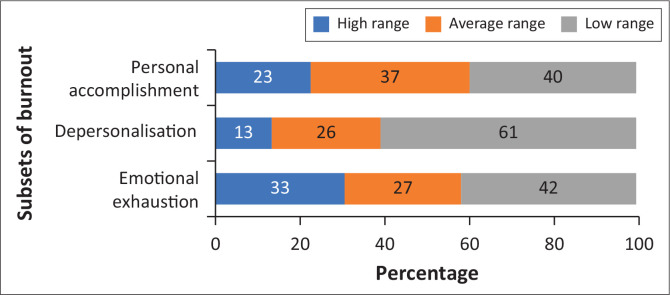
Burnout experienced by doctors.

A participant was considered to suffer from burnout when they scored within the high ranges in either the EE or DP burnout categories (see Table 1). The overall burnout rate for Mankweng Hospital was 33% and that of Pietersburg Hospital was 39%. The combined overall burnout rate for both hospitals was 36%. The mean EE for all participants was 21, the DP was 6 and the mean PA was 36 which means a moderate range burnout for EE and PA, while DP fell into the low range category. Of the participants, 49.3% (*n* = 74) presented with a high range of burnout in any of the three subscales, while 44% (*n* = 66) did not have a high range of burnout in any subscale. Worryingly, 13 (8.7%) participants presented with a high range of burnout in both EE and DP, while 26 (17.3%) participants showed a low range of burnout in all three subscales (see Figure 1 and Table 2 for more detail).

**TABLE 2:** Burnout experienced by doctors at both hospitals (*N* = 150).

The score range of burnout (MBI)	*n*	%
Participants with a high range in any of the three subscales	74	49.3
Participants with a high range in EE or DP	54	36.0
Participants with a high range in EE and in DP	13	8.7
Participants not scoring in high range in any of the three subscales	66	44.0
Participants with a low range in all three subscales	26	17.3
Participants with a high range in EE	46	30.7
Participants with a high range in DP	20	13.3
Participants with a high range in PA	35	23.3

MBI, Maslach burnout inventory; EE, emotional exhaustion; DP, depersonalisation; PA, personal accomplishment.

### Associations between burnout and demographic factors

**FIGURE 2:**
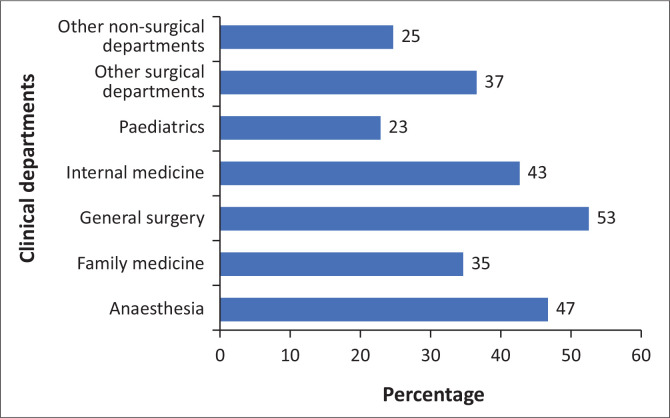
Burnout in doctors as per clinical department.

We found no statistically significant associations between burnout and various demographic covariates, including clinical departments. While burnout rates seemed to be higher in general surgery, anaesthesia and internal medicine, none of these differences were statistically significant (see Table 3 and Figure 2). Similarly, gender, age, marital status, length of practice, the average number of hours worked per week and participation in overtime did not have a statistically significant effect on burnout (see Table 4).

**TABLE 3:** Burnout experienced by doctors in the various clinical departments

Department	Total number of participants from that department	Total number with burnout from that department	Percentage of burnout for the individual department (%)
Anaesthesia	19	9	**47**
Family medicine	26	9	**35**
General surgery	15	8	**53**
Internal medicine	23	10	**43**
Paediatrics	35	8	**23**
Other surgical departments	16	6	**37**
Other non-surgical departments	16	4	**25**

**TABLE 4:** Comparison of burnout and demographic covariants.

Demographic covariants	Presence of burnout (from total of 150)			
	No burnout	Burnout present		*p*
		*n*	%	
**Gender**				0.212
Men	47	22	31.8	-
Women	49	32	39.5	-
**Age group (years)**				0.656
20–35	63	40	38.8	-
36–45	20	10	33.3	-
46–55	7	2	22.2	-
> 55	6	2	25.0	-
**Marital status**				0.131
Single	53	35	39.7	-
Married	43	17	28.3	-
Divorced	0	1	-	-
Separated	0	0	-	-
Widowed	0	1	-	-
**Length of practice (years)**				0.258
< 5	45	31	40.7	-
5–9.9	24	12	33.3	-
10–15	16	7	30.4	-
> 15	11	4	26.6	-
**Average hours per week (hour)**				0.183
< 30	1	1	50.0	-
31–50	38	14	26.9	-
51–70	47	30	38.9	-
71–90	7	8	53.3	-
> 90	3	1	25.0	-
**Overtime duties** ^†^^‡^				
**(YES)** Does perform overtime	94	51	35.2	0.200
**(NO)** Does not perform overtime	2	3	60.0	-
**Ranks**				
Intern doctors	34	22	39.0	-
Medical officers	34	22	39.0	-
Registrars	14	4	22.0	-
Consultants	14	6	30.0	-

† No burnout: Overtime duties *n* = 96;

‡ Burnout present: Overtime duties *n* = 54.

## Discussion

This study found a 36% prevalence of clinically significant burnout among doctors working at Mankweng and Pietersburg hospitals, in the Limpopo province of South Africa. Furthermore, 49.3% of doctors at these institutions scored high in at least one category of burnout which clearly indicates that prevention strategies are very important. No associations were found between burnout and demographic covariates.

Several South African studies on burnout among medical doctors have been conducted with varying results. Compared to the current study, some showed markedly higher levels of burnout, while others showed the reverse. Among rural doctors in the Western Cape, 81% of participants demonstrated high EE or DP scores.^7^ Similarly, in the Cape Metropole, level of burnout among public sector doctors was high at 76%.^8^ Conversely, a much lower overall burnout prevalence (26.3%) was shown among public sector doctors in Bloemfontein.^9^

Interestingly, this variation in burnout prevalence is a worldwide phenomenon. A review of 182 burnout studies including 109 628 individuals in 45 countries showed that overall burnout prevalence rates ranged from 0% to 80.5%.^10^ A similar trend was observed for the three burnout categories. Emotional exhaustion ranged from 0% to 86.2%, DP from 0% to 89.9% and PA prevalence from 0% to 87.1%. While most of the studies included in this review used the MBI as a measuring tool, these wide ranges have been ascribed to variation in the criteria used to define and measure overall burnout. Considering there are at least 142 unique definitions for meeting the criteria of overall burnout or burnout within a subscale, this is not surprising.^10^ Unfortunately, this lack of consensus among researchers makes between study comparisons problematic.^10^

In a review of burnout prevalence and associated factors in the UK, burnout scores for EE ranged from 31% to 54.3%, DP levels were between 17.4% and 44.5%, while PA ranged from 6% to 39.6%. In this study, general practitioners and consultants were shown to have the highest scores, and contributing factors to burnout were low job satisfaction, overload and long working hours. Psychiatric morbidity both contribute to the development of burnout and is a consequence of burnout.^14^

In light of the above, the 36% burnout prevalence found at Pietersburg and Mankweng hospitals seems to fall within the lower middle range compared to studies from South Africa and internationally.^7,8,9,10,14^ However, this assumption should be viewed with caution. While it seems like the prevalence of burnout varies a lot from place to place, as the work circumstances may differ greatly, the reasons for this variation might also be because of definitions and criteria as opposed to burnout itself. The possibility therefore arises that we may, in fact, not be comparing apples with apples after all.

This study did not demonstrate any statistically significant associations between burnout and demographic factors, a finding noted by other research as well.^10,15^ Furthermore, genetic factors seem to only explain 33% of differences in burnout symptoms, with environmental influences believed to play the bigger role.^16^ The rank and work experience of doctors have been investigated by researchers as contributing factors towards burnout. High burnout rates among junior doctors have been extensively discussed in the literature and is ascribed to the increased clinical responsibility often unsupervised or alone.^7,17^ In Australia, many medical graduates feel unprepared for clinical practice. They are required to manage acutely ill patients and handle stressful workloads. The psychological stress, mental illness and general dissatisfaction with their career and life contribute to the above. The lack of support from senior staff and ambiguity of future career progression are also common concerns.^15^ This study did not demonstrate any statistically significant differences in burnout between rank or work experience; however, this is likely because of the small sample size of some of the sub-groups. More research is needed in similar settings before drawing any definitive conclusions.

## Limitations of the study

Our final sample size of 150 was less than the expected 194 as per calculated sample. A further limitation is that participants were recruited conveniently, and consequently, the results are not generalisable. The small number of doctors in some of the sub-groups limited the reliability of between-group comparisons. Furthermore, the sample comprised doctors who were present at departmental meetings and available to fill out the questionnaires. This implies convenience sampling and limits the generalisability of the results – it is very possible that the doctors who happened to be absent from the weekly continuing medical education meetings might have been the ones who were most burned out.

## Conclusion

While a burnout prevalence of 36% at Pietersburg and Mankweng hospitals seems to fall within the lower middle range of what has been reported in South Africa and elsewhere, this must be viewed with caution.^7,8,9,10,14^ Research on burnout among medical doctors has shown a large degree of variation, most likely because of extraneous factors.

In keeping with the literature, our study showed no associations between sociodemographic factors and burnout, which either suggests that the cause of burnout should be sought elsewhere, or simply that the phenomenon of burnout is complex and multifactorial in origin.

There is too much variation in the criteria of burnout among different studies, making comparisons difficult. More studies are needed to standardise the measurement of burnout.
